# Zika virus causes supernumerary foci with centriolar proteins and impaired spindle positioning

**DOI:** 10.1098/rsob.160231

**Published:** 2017-01-18

**Authors:** Benita Wolf, Fodé Diop, Pauline Ferraris, Sineewanlaya Wichit, Coralie Busso, Dorothée Missé, Pierre Gönczy

**Affiliations:** 1 Swiss Institute for Experimental Cancer Research (ISREC), School of Life Sciences, Swiss Federal Institute of Technology Lausanne (EPFL), 1015, Lausanne, Switzerland; 2 Laboratoire MIVEGEC, UMR 224 IRD/CNRS/UM1, 34394 Montpellier, France

**Keywords:** Zika virus, dengue virus, microcephaly, centrosome, spindle positioning

## Abstract

Zika virus (ZIKV) causes congenital microcephaly. Although ZIKV can impair cell cycle progression and provoke apoptosis, which probably contributes to disease aetiology through depletion of neural progenitor cells, additional cellular mechanisms may be important. Here, we investigated whether ZIKV infection alters centrosome number and spindle positioning, because such defects are thought to be at the root of inherited primary autosomal recessive microcephaly (MCPH). In addition to HeLa cells, in which centrosome number and spindle positioning can be well monitored, we analysed retinal epithelial cells (RPE-1), as well as brain-derived microglial (CHME-5) and neural progenitor (ReN) cells, using immunofluorescence. We established that ZIKV infection leads to supernumerary foci containing centriolar proteins that in some cases drive multipolar spindle assembly, as well as spindle positioning defects in HeLa, RPE-1 and CHME-5 cells, but not in ReN cells. We uncovered similar phenotypes in HeLa cells upon infection with dengue virus (DENV-2), another flavivirus that does not target brain cells and does not cause microcephaly. We conclude that infection with *Flaviviridae* can increase centrosome numbers and impair spindle positioning, thus potentially contributing to microcephaly in the case of Zika.

## 1. Background

Zika virus (ZIKV) is a member of the *Flaviviridae* family of viruses that can infect human beings [1]. Although adults infected by ZIKV usually suffer from mild clinical symptoms, numerous cases of congenital microcephaly, in Brazil in particular, transformed the Zika threat into a worldwide public health emergency [2,3]. Intrauterine infections with other viruses, including rubella virus, herpes simplex virus and cytomegalovirus, can also cause microcephaly, but these viruses impair the development of other organs in addition to that of the brain [4,5]. By contrast, the impact of ZIKV on the fetus is essentially limited to causing a small brain [6,7]. Intriguingly, such a brain-restricted impact is also observed in primary autosomal recessive microcephaly (MCPH), the most prevalent genetic cause of congenital microcephaly [8]. Whether ZIKV-mediated infection causes similar cellular phenotypes as those observed in MCPH is not clear.

Most genes mutated in MCPH encode centrosomal proteins [8–10]. Centrosomes are the primary microtubule organizing centre (MTOC) of animal cells and contain centrioles surrounded by pericentriolar material (PCM) [11,12]. The centrosome notably directs cell polarity during interphase and bipolar spindle assembly during mitosis. In tissue culture HeLa cells, depletion of MCPH gene products can lead to the assembly of a bipolar spindle that is mispositioned with respect to a reference substratum such as a fibronectin-coated surface [13,14]. In the developing mouse brain, such spindle mispositioning results in the depletion of neural progenitor cells [15,16], raising the possibility that spindle positioning defects may contribute to disease in microcephaly patients. Furthermore, experimental induction of supernumerary centrioles in the developing mouse brain results in the assembly of multipolar spindles, leading to aneuploid daughter cells that undergo apoptosis and thereby cause microcephaly [17].

## 2. Results and discussion

We set out to investigate whether ZIKV infection of cultured human cells leads to abnormal centrosome numbers and spindle positioning defects. We conducted initial experiments in HeLa cells, which have been utilized previously to analyse these processes in a range of other experimental settings. We first tested whether HeLa cells could be infected by different strains of ZIKV. Phylogenetic analysis shows that ZIKV originated from Uganda, from where it followed two major lines of viral evolution, one in Africa and one in Asia, the latter then yielding the Brazilian strain causing the current epidemic outbreak [1]. Therefore, we included viral strains of both African (strains Arb 15076 and Hd 78788) and Asian (Pf-25013-18) origins. As reported in electronic supplementary material, figure 1*a*, using immunofluorescence analysis of flavivirus envelopes (4G2) as a readout, we found that HeLa cells can indeed be infected by these three strains of ZIKV.

We restricted further analysis to 4G2 positive cells (electronic supplementary material, figure S1*b*), even though other cells exhibited clear signs of infection, such as cell membrane bags or cytoplasmic vacuoles (electronic supplementary material, figure S1*c*). Moreover, we excluded cells that were polyploid or that appeared compromised judging from their DNA (electronic supplementary material, figure S1*c*,*d*). Data from 24 and 48 h post-infection were pooled because the phenotypes of infected cells at the two time points were indistinguishable (see electronic supplementary material, table S1 for all experimental conditions and outcome).

**Figure 1.**
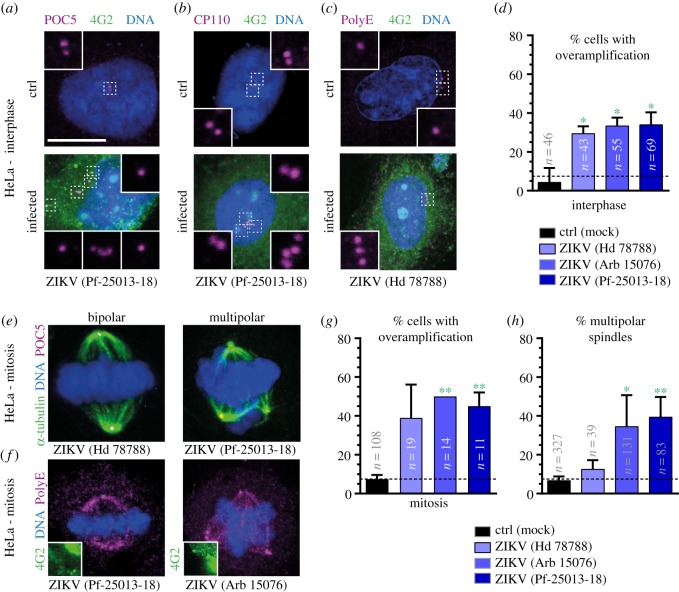
ZIKV infections lead to supernumerary foci of centriolar proteins and multipolar spindle assembly in HeLa cells. (*a*–*c*) Immunofluorescence images of mock-treated (ctrl) and ZIKV-infected HeLa cells in interphase, stained with antibodies against the centriolar proteins POC5 (*a*), CP110 (*b*) or PolyE (*c*) (all shown in purple), in combination with antibodies against the viral marker 4G2 (green). In this and other figures, DNA is shown in blue, scale bars correspond to 10 µm and insets are three times magnified views of select planes in the indicated regions from the lower magnification image (unless stated otherwise); the specific ZIKV strain is indicated in each case below the images. (*d*) Average percentage of cells (±s.d. of three biological replicates for ctrl and two biological replicates for ZIKV) exhibiting supernumerary foci of centriolar proteins, pooling the data from CP110, POC5 and PolyE stainings (*n*: total number of scored cells; see electronic supplementary material, table S1 for details). Unpaired two-tailed, Student's *t*-test comparing to control conditions: *p < 0.05. (*e*,*f*) Immunofluorescence images of mitotic HeLa cells infected by ZIKV and stained with antibodies against α-tubulin to mark spindle microtubules (green) and POC5 to mark centrioles (purple) (*e*), or against PolyE, which also label spindle microtubules close to the spindle poles (purple) and 4G2 (green) as an infection marker (*f*). Shown is a partial view of the 4G2 staining so as to not obliterate the DNA and centriolar signals. (*g*) Average percentage of mitotic cells (±s.d. of three biological replicates for ctrl and two biological replicates for ZIKV) exhibiting supernumerary centriolar foci. Note that the average for both datasets with ZIKV (Arb 15076) is 50% of cells, thus explaining the lack of standard deviation (n: total number of scored cells, see electronic supplementary material, table S1 for details). (*h*) Average percentage of mitotic cells (±s.d. of three biological replicates for ctrl and two biological replicates for ZIKV, data independent from that in figure 1*g*) exhibiting multipolar metaphase spindles. Unpaired two-tailed Student's *t*-test, comparing to control conditions: *p < 0.05, **p < 0.01.

To monitor centrosome numbers, we used immunofluorescence of cells stained with antibodies against 4G2, to ascertain infection status, as well as with antibodies against the centriolar markers POC5, CP110 or polygluatmylated tubulin (PolyE). Both POC5 and CP110 mark the distal part of centriolar cylinders [18,19]. Depending on the cell cycle stage, there are normally 2 or 4 foci of POC5 or CP110 per cell (figure 1*a*,*b*, ctrl) [11]. PolyE labels centrioles as they mature [20], such that two foci are present throughout the cell cycle (figure 1*c*, ctrl). We began our analysis with interphase cells. We found that infection of HeLa cells with ZIKV led to a significant augmentation of foci harbouring centriolar markers, from approximately 5% in control conditions to approximately 30–35%, depending on the sample, in ZIKV-infected interphase cells (figure 1*a*–*d*). Using nuclear area as a proxy for cell cycle stage [21], we established that supernumerary foci with centriolar proteins were apparent in interphase cells at any stage of the cell cycle (electronic supplementary material, figure 2*a*).

We next set out to address whether supernumerary centriolar protein foci can be detected during mitosis in ZIKV-infected HeLa cells. As shown in figure 1*e*–*g*, we found this to be the case. Furthermore, we found that these supernumerary foci had microtubule organizing capacity, because ZIKV-containing cells frequently assembled a multipolar spindle during mitosis (figure 1*e*,*f*,*h*). Such multipolar figures are expected to yield aneuploid daughter cells and result in cell death, as upon the presence of supernumerary centrioles in the developing mouse brain [17]. Taken together, these findings lead us to conclude that infection with ZIKV leads, directly or indirectly, to an augmentation of centriole numbers in HeLa cells. These results are in line with observations in human neuroepithelial stem cells and radial glia [22], as well as in neural progenitor cells in the mouse [23], which suggested the presence of supernumerary centrosomes upon ZIKV infection.

HeLa cells are derived from a cervical cancer and harbour an integrated human papilloma virus (HPV) viral genome [24]. To explore whether the phenotypes reported above stem merely from cells harbouring HPV, we expanded our analysis to untransformed human retinal epithelia RPE-1 cells, which are immortalized by hTert. Moreover, as the pathologic consequences of ZIKV infection in human beings are restricted primarily to the developing brain, we also tested the SV40-transformed microglial cell line of embryonic origin CHME-5, as well as the v-myc transformed neural progenitor cell line ReN. The consequences of ZIKV infection on the number of foci with centriolar markers during mitosis and on spindle positioning were analysed in these three cell lines 48 h following infection by the Polynesian strain (Pf-25013-18), which is closest to the Brazilian strain (see electronic supplementary material, figure S1*e* for infection rates in those three cell lines and table S1 for all experimental conditions and outcome).

**Figure 2.**
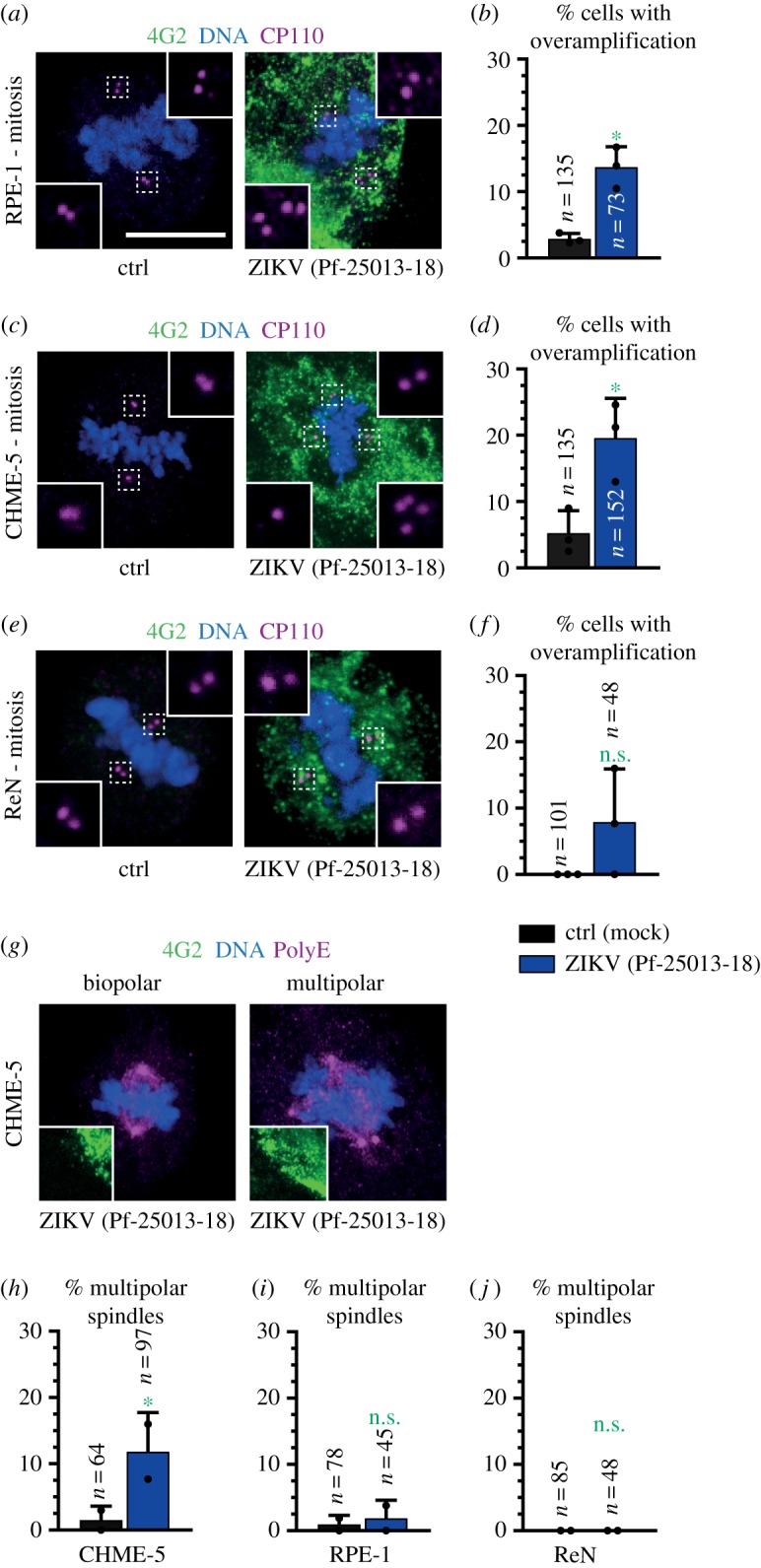
ZIKV infections lead to supernumerary foci of centriolar proteins and multipolar spindle assembly in CHME-5 cells and RPE-1 cells. (*a*,*c*,*e*) Immunofluorescence images of mitotic RPE-1 (*a*), CHME-5 (*c*) and ReN (*e*) cells either mock-treated (ctrl) or subjected to ZIKV infection (strain Pf-25013-18), and stained with antibodies against CP110 to mark centrioles (red) and 4G2 to mark viral infection (green). (*b*,*d*,*f*) Average percentage of mitotic RPE-1 (*b*), CHME-5 (*d*) and ReN (*f*) cells exhibiting supernumerary centriolar foci of CP110 or POC5 (±s.d. of three coverslips—two stained for CP110 and one stained for POC5; see electronic supplementary material, table S1; *n*; total number of cells scored). Unpaired two-tailed Student's *t*-test, comparing to control conditions for each cell line: *p < 0.05. (*g*) Immunofluorescence images of mitotic CHME-5 cells subjected to ZIKV infection and stained with antibodies against PolyE, which label also spindle microtubules close to the spindle poles (purple) and 4G2 as an infection marker (green). (*h*–*j*) Average percentage of mitotic RPE-1 (*i*), CHME-5 (*h*) and ReN (*j*) cells (±s.d. of two technical replicates for each condition) exhibiting multipolar metaphase spindles. Unpaired two-tailed Student's *t*-test, comparing to control conditions for each cell line: *p < 0.05, **p < 0.01. *n*: total number of metaphase cells scored.

Staining with antibodies against CP110 or POC5 established that RPE-1 and CHME-5 cells also exhibited supernumerary foci of centriolar proteins (figure 2*a*–*d*). Probably as a consequence, ZIKV-infected CHME-5 cells could assemble a multipolar spindle during mitosis (figure 2*g*,*h*), whereas this was not the case in ZIKV-infected RPE-1 cells (figure 2*i*), perhaps because these cells possess a robust ability to cluster supernumerary centrioles into two spindle poles [25,26]. There was no statistically significant increase in foci of centriolar proteins in ReN cells (figure 2*e*,*f*,*j*), which is surprising considering the recent observations in related human neuroepithelial stem cells and radial glia, as well as in neural progenitor cells in the mouse [22,23]. Resolving the root of this difference will require further work. Overall, we conclude that ZIKV infection leads to supernumerary foci of centriolar proteins in transformed HeLa and microglial cells (CHME-5), as well as in non-transformed retinal epithelial cells (RPE-1).

**Figure 3.**
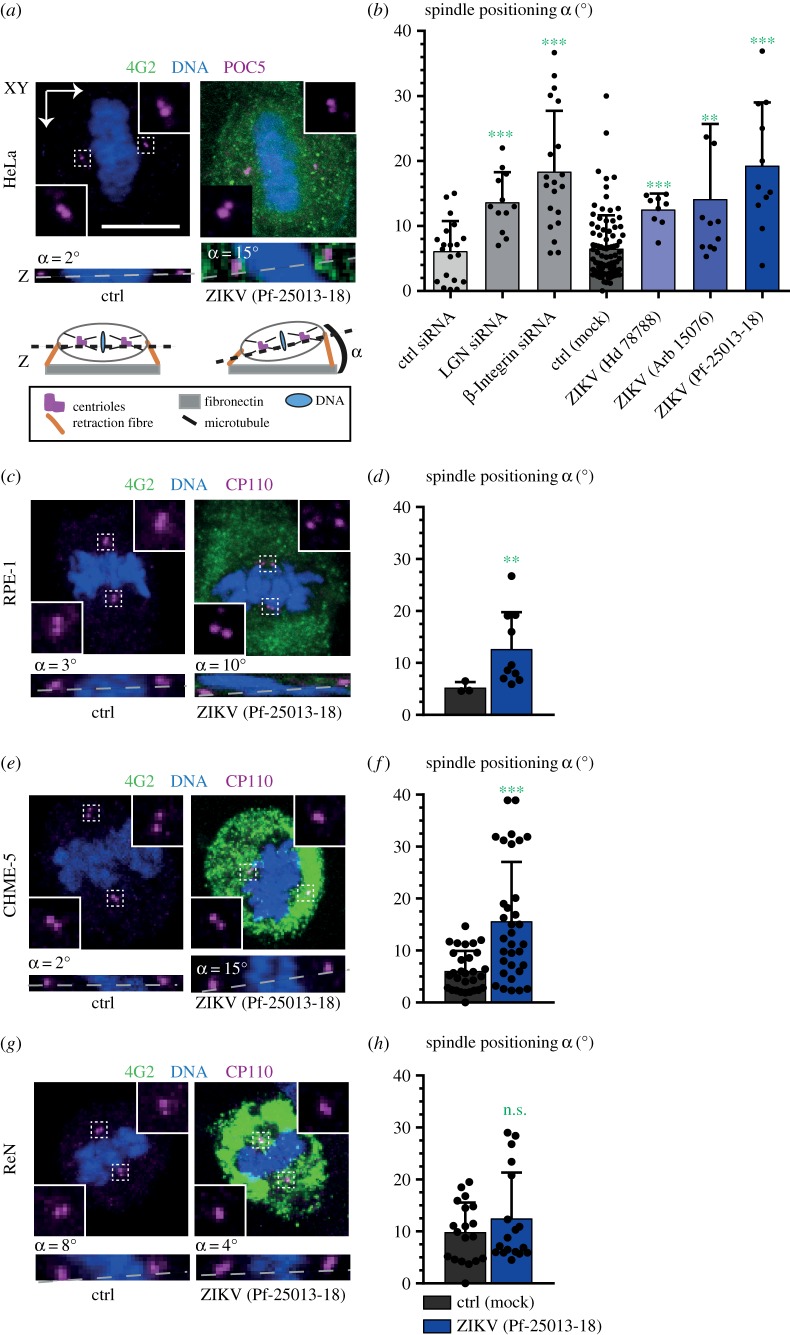
ZIKV impairs spindle positioning. (*a*,*b*) Spindle positioning assay in HeLa cells. (*a*) Immunofluorescence images of mock-treated (ctrl) and ZIKV-infected HeLa cells stained with antibodies against POC5 (purple) and 4G2 (green). *Z*-stacks of 0.4 µm-high confocal sections were imaged between the two spindle poles; shown are projections of relevant planes (top, *XY*), as well as resliced sections (bottom, *Z*), which were used to determine the angle *α* of the spindle axis with respect to the substratum, as schematized below. (*b*) Average spindle positioning angle for indicated siRNA treatments (three left-most bars, two biological replicates) and indicated infection conditions (±s.d. of three (mock) or two (ZIKV) biological replicates, four right-most bars; see electronic supplementary material, table S1 for details). Mann–Whitney *U*-test, comparing to control conditions: ***p < 0.001, **p < 0.01. (*c*,*e*,*g*) Immunofluorescence images of mock-treated (ctrl) and ZIKV-infected RPE-1 (*c*), CHME-5 (*e*) and ReN (*g*) cells stained with antibodies against CP110 (purple) and 4G2 (green); spindle-positioning analysis was performed as described above for HeLa cells. (*d*,*f*,*h*) Average spindle positioning angle (±s.d. of three technical replicates for each condition) of mock versus ZIKV-infected cells for different cell types. Mann–Whitney *U*-test, comparing to control conditions: ***p < 0.001, **p < 0.01. See electronic supplementary material, table S1 for details.

We then assayed spindle positioning in infected cells that assembled a bipolar spindle. To this end, cells were plated on fibronectin-coated coverslips, which provide a planar substratum that normally directs spindle positioning parallel to it (figure 3*a*) [27]. Upon siRNA-mediated depletion of components critical for spindle positioning, such as LGN or β-integrin [27], the spindle tends not to be positioned parallel to the substratum, but instead at an angle from it (figure 3*b*). Importantly, measurements of the angle between the bipolar mitotic spindle and the substratum revealed striking defects in spindle positioning upon infection of HeLa cells with ZIKV (figure 3*a*,*b*). Moreover, we found that spindle positioning defects were not correlated with increases in the number of foci with centriolar proteins, suggesting independent mechanisms (electronic supplementary material, figure S2*b*). Next, we measured spindle positioning in RPE-1, CHME-5 and ReN cells. As shown in figure 3*c*–*h*, this analysis uncovered spindle-positioning defects in both RPE-1 and CHME-5 cells, but not in ReN cells. Overall, we conclude that ZIKV infection can cause impaired spindle positioning.

**Figure 4.**
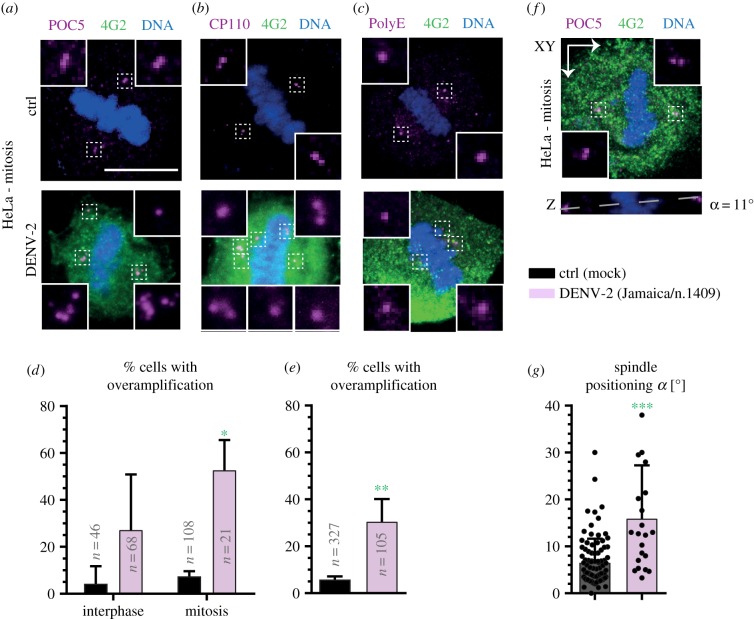
DENV-2 infection in HeLa cells leads to supernumerary foci of centriolar proteins, multipolar spindle assembly and impaired spindle positioning. (*a*–*c*) Immunofluorescence images of mock-treated (ctrl) and DENV-2-infected HeLa cells in mitosis, stained with antibodies against the centriolar proteins POC5 (*a*), CP110 (*b*) or PolyE (*c*) (all shown in purple), in combination with antibodies against the viral marker 4G2 (green). (*d*) Average percentage of interphase and mitotic cells (±s.d. of three biological replicates for both ctrl and DENV-2) exhibiting supernumerary centriolar foci, pooling the data from CP110, POC5 and PolyE stainings. Unpaired two-tailed, Student's *t*-test, comparing to control conditions: *p < 0.05 (see electronic supplementary material, table S1 for details). Note that control cells were the same for ZIKV and DENV-2 experiments because they were performed at the same time. (*e*) Average percentage of mitotic cells (±s.d. of three biological replicates for both ctrl and DENV-2) with multipolar spindles. Unpaired two-tailed Student's *t*-test, compared to control conditions: **p < 0.01. (*f*) Immunofluorescence image of DENV-2-infected HeLa cells stained with antibodies against POC5 (purple) and 4G2 (green); spindle-positioning analysis was performed as described in figure 3*a*. (*g*) Average spindle-positioning angle (±s.d. of three biological replicates for both ctrl and DENV-2) for ctrl and DENV-2-infected cells. Mann–Whitney *U*-test, compared to control conditions: ***p < 0.001.

In order to test whether the above phenotypes are specific to ZIKV or instead represent more general characteristics of infection by *Flaviviridae*, we also tested the impact of dengue virus (DENV, strain DENV-2) in HeLa cells. Although the two viruses are closely related and can bind to similar cell surface receptors [28–30], DENV is not neurotrophic and therefore does not cause microcephaly [31,32]. After confirming that HeLa cells can be infected with DENV-2 virus (electronic supplementary material, figure S1*a*), we conducted analyses similar to those performed for ZIKV. As shown in figure 4*a*–*d*, we likewise found an increase in the number of centriolar foci in both interphase and mitotic HeLa cells upon DENV infection. As in the case of ZIKV, we also found an augmentation in foci of centriolar proteins irrespective of cell cycle stage upon DENV infection (electronic supplementary material, figure S2*c*). Moreover, DENV infection led to mitotic cells undergoing multipolar spindle assembly and impaired spindle positioning (figure 4*e*–*g*), independently of the number of centrioles (electronic supplementary material, figure S2*d*).

In conclusion, we have shown that ZIKV infection leads to the presence of supernumerary foci of centriolar proteins as well as to defects in spindle positioning in HeLa, RPE-1 and CHME-5 cells. DENV has similar effects in HeLa cells. Viral infection with ZIKV or DENV also decreased cell numbers in all cell lines (data not shown), in line with the notion that ZIKV and DENV infections lead to cell death [23,33]. It will be interesting to address whether spindle-positioning phenotypes are observed in animal models of ZIKV infection, as well as in affected human fetuses. There are other documented cases of viruses impacting centrosomes. For instance, mutated pre-hepatitis B virus particles can lead to centriole amplification through increased calcium entry [34], whereas human T cell leukemia virus type-1 (HTLV-1) causes abnormal centrosome fragmentation through the targeting of Ran-binding protein-1, leading to supernumerary centrioles [35]. Further work is needed in the case of the *Flaviviridae* to elucidate the mechanisms through which ZIKV and DENV alter the centriole duplication cycle. Besides the possibility that these phenotypes reflect a general non-specific cellular response, which appears unlikely given that they are not observed in ReN cells, potential mechanisms include the premature licensing of centriole formation, as observed in cells held in the G2 phase of the cell cycle [36], or overexpression of components driving centriole formation such as Plk4 or HsSAS-6 [37,38].

Together with previously established consequences of ZIKV on cell cycle progression and cell survival [39–41], the occurrence of multipolar and mispositioned spindles could conceivably contribute to intrauterine microcephaly caused by ZIKV infection. These findings offer a novel illustration of the intricate cellular relationships between viruses and their hosts, and should lead to a better understanding of viral pathological mechanisms.

## 3. Material and methods

### 3.1. Cells and viruses

HeLa cells were purchased from ECACC (ECACC 93021013), RPE-1 cells from ATCC (Manassas, VA; CRL-4000). CHME-5 cells were a kind gift of Ali Amara [42], ReN cells were a gift of Chiara Sartori, who originally purchased them from EMD Millipore (SCC008). Vero cells were used to titrate viruses. HeLa, RPE-1, CHME-5 and Vero cells were cultured in high-glucose DMEM medium with GlutaMAX (Invitrogen) or in DME-F12 (Vero cells, Invitrogen) medium, each supplemented with 10% FCS, in a humidified 5% CO_2_ incubator at 37°C. ReN cells were cultivated in DMEM F12 (Gibco) containing Glutamax (2 mM), B-27 stem cell supplement (Gibco) 1U ml^−1^ Heparin (Sigma), 20 ng ml^−1^ bFGF (Peprotech) and 20 ng ml^−1^ EGF (Peprotech). ReN cells were grown on Laminin (0.7 ug cm^−2^, Sigma) coated cell culture ware or coverslips.

Propagation of the ZIKV strains and DENV-2 was achieved using *A. albopictus* C6/36 cells having undergone limited passages. C6/36 cells were grown in DMEM supplemented with 10% FCS and cultivated at 28°C.

The following viruses were used: the clinical isolate Pf-25013-18 [30]; the Hd 78788 strain obtained from a patient in Senegal during routine surveillance in 1991 [43]; the Arb 15076 strain isolated from *A. africanus* in central African Republic [44]; and the DENV-2 Jamaica/N.1409 strain (GenBank accession no. M20558.1).

We noticed upon immunofluorescence analysis that HeLa cells were probably infected with mycoplasma. This is unlikely to have influenced the outcome of the experiments because the same batch of HeLa cells was analysed for control and infected samples. Moreover, we tested RPE-1, CHME-5 and ReN cells, which were all mycoplasma-free (GATC Biotech report no:737054).

### 3.2. Infection of cells

For infection, cells were seeded on glass- or fibronectin-coated coverslips in six-well culture plates at a density of 100 000 cells per well. Then, 24 h after seeding, cells were rinsed once with phosphate-buffered saline (PBS), and viruses, which were diluted to 5–10 multiplicity of infection (MOI), were added to the cells. Cells were then incubated for 2 h at 37°C with gentle agitation every 30 min. Next, the inoculum was removed and cells were washed twice with PBS. Culture medium was added to each well, and cells were incubated at 37°C and 5% CO_2_ for the duration of the experiment. As a control, cells were incubated with the culture supernatant from uninfected C6/36 cells, referred to as mock treated -or control- cells.

### 3.3. Immunofluorescence, imaging and analysis

For immunofluorescence, cells were fixed in −20°C methanol for 7–10 min and washed in PBS-0.05% Triton X-100 (PBST). After blocking in 1% BSA in PBST for 1 h, cells were incubated with primary antibodies at room temperature for 4 h. After three washes in PBST for 5 min each, cells were incubated with secondary antibodies for 1 h at room temperature, stained with 1 μg ml^−1^ Hoechst 33342 (Sigma-Aldrich), washed three times 5 min in PBST, and mounted. Primary antibodies were 1 : 200 mouse anti-4G2 (MAB10216, Millipore), 1 : 1000 rabbit anti-PolyE, (pAb IN105 Adipogen), 1 : 1000 rabbit anti-CP110 (127801-AP; ProteinTech Europe), 1 : 1000 rabbit anti-POC5 [18] and 1 : 200 mouse anti-α-tubulin (DM1α, Sigma-Aldrich). Secondary antibodies were Alexa Fluor 488-coupled anti-mouse and Alexa Fluor 568-coupled anti-rabbit, both used at 1 : 1000 (Life Technologies).

Images were acquired with a 63×, NA 1.0 oil objective on a confocal microscope (LSM 700; Carl Zeiss, 526 × 526 pixel minimal resolution), equipped with a charge-coupled device camera (AxioCam MRm; Carl Zeiss), then processed and analysed in ImageJ (National Institutes of Health). Relevant *z*-planes are shown as maximum intensity projections; in insets, *z*-planes, relevant for this particular centrosome are used for projections, while in overview images *z*-planes relevant for all centrosomes of the cell are used for projections. Data were analysed using Excel and statistics performed with GraphPad.

For analysis of centriolar protein foci and of spindle positioning in HeLa cells, confocal images (using a 63× Plan-Apochromat, NA 1.4 objective) were taken of 154 control cells and 211 cells infected with one of the three viral strains in two biological replicates for ZIKVs and three replicates for controls as well as 90 cells infected with DENV-2 virus in three biological replicates. For analysis of centriolar protein foci and of spindle positioning in RPE-1, CHME-5 and ReN cells, confocal images (using a 63× Plan-Apochromat, NA 1.4 objective) were taken of cells on fibronectin-coated coverslips stained with CP110 and 4G2 antibodies (RPE-1: 39 ctrl and 36 infected cells, CHME-5: 40 ctrl and 41 infected cells, ReN: 19 ctrl and 18 infected cells). Further analysis of centriolar protein foci was conducted on cells plated on glass coverslips, stained with either CP110 and 4G2 or POC5 and 4G2 antibodies, using a 100× objective (Plan-Apochromat, NA 1.4) at a wide-field microscope (RPE-1: 96 ctrl, 37 infected, CHME-5: 95 ctrl, 111 infected, ReN: 75 ctrl and 35 infected cells). Data from 24 and 48 h post-infection were pooled because the phenotypes of infected cells at the two time points were indistinguishable for HeLa cells; only 48 h time point following infection was analysed in the case of RPE-1, CHME-5 and ReN cells (see electronic supplementary material, table S1 for all experimental conditions and outcome).

To determine whether the spindle was bipolar or multipolar, metaphase cells stained with PolyE or α-tubulin were analysed using a 63× (Plan-Apochromat, NA 1.4) objective at a wide-field microscope. This analysis was performed on all cells, independent of 4G2 status, such that the actual fraction of infected cells with a multipolar spindle may be higher than what is reported. For HeLa cells, data from four (five for ctrl) independent coverslips originating from two biological replicates for ZIKV and three biological replicates for ctrl and DENV-2 with indicated number of metaphase cells (*n*) were analysed (*n*; control: 74, 101, 49, 52, 51; ZIKV (Hd 78788): 8, 10, 18, 3; ZIKV (Arb 15076): 54, 30, 20, 27; ZIKV (Pf-25013-18): 17, 15, 37, 14; DENV-2: 13, 26, 20, 21, 25). For RPE-1, CHME-5 and ReN cells, data from two technical replicate with indicated numbers of metaphase cells (*n*) were analysed (*n*; RPE-1 ctrl: 53, 25; RPE-1 ZIKV (Pf-25013-18): 19, 26; CHME-5 ctrl: 33, 31; CHME-5 ZIKV (Pf-25013-18): 39, 58; ReN ctrl: 50, 35; ReN ZIKV (Pf-25013-18): 23, 25).

Nuclear areas (in square micrometres) were measured from maximum intensity projections of confocal images using ImageJ.

We compared two conditions (ctrl versus infection) at a time using unpaired, two-tailed Student's *t*-test for samples of unequal variances and sample sizes (also known as Welch's test). If normality test failed for one of the groups within a comparison, Mann–Whitney-*U* test was applied as stated in figure legends for each case.

### 3.4. siRNA treatment

For siRNA experiments, approximately 100 000 cells were seeded on fibronectin-coated coverslips in six-well plates and transfected with 20 nM of validated stealth siRNAs (Qiagen) against β1-Integrin (accession number NM-033669, nucleotide 167–189), LGN (5′-GAACUAACAGCACGACUUA-3′, 5′-CUUCAGGGAUGCAGUUAUA-3′, 5′-ACAGUGAAAUUCUUGCUAA-3′, 5′-UGAAGGGUUCUUUGACUUA-3′) or scrambled siRNA (SI03650318) as per the manufacturer's instructions. In brief, 2.5 µl of 20 µM siRNA in 250 µl Optimem and 7.5 µl Lipofectamin RNAi max (Invitrogen) in 250 µl Optimem were incubated in parallel for 5 min, mixed for 20 min and then added to 2 ml of medium per well; cells were incubated for 48 h before fixation and analysis.

### 3.5. Spindle positioning assay

To monitor spindle positioning, cells were grown on coverslips uniformly coated with fibronectin (Neuvitro, GG-22-fibronectin). After fixation and immunofluorescence, the angle of the metaphase spindle with respect to the fibronectin substratum was determined as described [27]. In brief, cells were stained with POC-5, CP110 or PolyE antibodies to mark spindle poles and counterstained with 1 μg ml^−1^ Hoechst 33342 (Sigma-Aldrich) to mark chromosomes. Stacks of confocal images 0.4 μm apart were acquired. ImageJ was used to determine distances between spindle poles in *x, y* and *z*, and the angle *α* calculated using a custom-made ImageJ macro. Lower-most positions of spindle poles were used to draw the connecting line between them (see schematic in figure 3*a*). If centrioles within a given spindle pole laid in different planes of the *z*-stack, the angle was measured between the ones that were closest, to err on the side of caution.

## Supplementary Material

Zika, centrosomes and mitotic spindles - Electronic Supplementary Material

## Data accessibility

The datasets supporting this article have been uploaded as part of the electronic supplementary material.
