# Supplementary material for: Zika virus causes supernumerary foci with centriolar proteins and impaired spindle positioning
Source: Open Biol. 2017 Jan 18;7(1):160231. doi: 10.1098/rsob.160231 (PMC5303270; doi:10.1098/rsob.160231)
Supplement: Zika, centrosomes and mitotic spindles - Electronic Supplementary Material [file rsob160231supp1.pdf]

Electronic Supplemental Material
Open Biology
DOI: 10.1098/rsob.2016

Zika virus causes supernumerary foci with centriolar proteins and impaired spindle positioning

Benita Wolf, Fodé Diop*, Pauline Ferraris*, Sineewanlaya Wichit*, Coralie Busso, Dorothée Missé, Pierre Gönczy

* These authors contributed equally to the work.

Correspondence: Pierre.Gonczy@epfl.ch

Supplemental Figure 1

HeLa cells can be infected with ZIKV and DENV-2

(A) Percentage of 4G2-positive HeLa cells 48 hours post infection (p.i.) with indicated viruses. Data points correspond to scored coverslips (2-3 per biological replicate; n: number of cells scored; mock: 863, ZIKV (Hd 78788): 364, ZIKV (Arb 15076): 421, ZIKV (Pf-25013-18): 961).

(B) Immunofluorescence analysis with antibodies against 4G2 (green) to detect flaviviruses in interphase and mitotic cells; DNA is shown in blue. We included in the analysis cells that showed clear signs of viral infection as judged by 4G2 staining (ZIKV n= 199, DENV-2 n=91 cells, see images) or cytoplasmic 4G2 levels being >3 s.d. higher than the average intensity of uninfected control cells (12 cells, not shown).

(C) Categories of cells that were likely infected but excluded from the analysis. These are, from left to right: cells with cytoplasmic levels of 4G2 signal that were higher than the average of uninfected control cells, but <3 s.d. than that average; cells with a peripheral membrane "bag" that is 4G2 positive (arrow); cells with large vacuoles in the cytoplasm (arrow); polyploid cells; compromised cells. All scale bars: 10µm.

(D) Example of cells showing seemingly a normal DNA staining (left) versus what we refer to as a "compromised" phenotype. Cells were stained with anti-4G2 antibody (green), DNA is shown in blue.

(E) Percentage of 4G2-positive RPE-1, CHME-5 and ReN cells 48 hours post infection (p.i.) with ZIKV (Pf-25013-18) virus. Data points correspond to scored coverslips. (n: number of cells scored; RPE-1 mock: 617; RPE-1 ZIKV (Pf-25013-18): 526; CHME-5

mock: 1006; CHME-5 ZIKV (Pf-25013-18); 1092, ReN mock: 440; ReN ZIKV (Pf-25013-18): 900).

Supplemental Figure 2:

Centrosome amplification is apparent in different stages of the cell cycle, and is independent from a spindle positioning phenotype in ZIKV and DENV-2 infected cells

(A, C) Measurements of nuclear area as proxy of cell cycle stage in interphase ctrl and ZIKV (**A**, all three strains combined) or DENV-2 (**C**) infected HeLa cells that were included in the study (except 13 that were imaged with lower resolution), showing no differences between cells with ≤4 foci of centriolar proteins (n= 141 (**A**), n=72 (**C**)) and cells with >4 such foci (supernumerary, n=55 (**A**) n=17 (**C**)) (Kolmogorov-Smirnov test, p=0.5040 (**A**), p=0.3805 (**C**)).

(**B, D**) Comparison of average (± s.d. of three biological replicates for ctrl and DENV-2, two biological replicates for ZIKV) spindle positioning angles of cells with normal counts of centriolar protein focis (grey, n=9 (**B**), n=8 (**D**)) and cells with supernumerary foci (>4 foci, green, n=18 (**B**), n=11 (**D**)), showing no significant difference (unpaired two-tailed Student’s t-test, p>0.5) in ZIKV (**B**, Hd 78788, Arb 15076, Pf-25013-18) or DENV-2

Table S1

Overview of all analyzed cells. Each row corresponds to an experimental condition and contains data pooled of 24h and 48h p.i. experiments.

Column headings indicate from left to right: [coverslip]; cell type used [cell]; viral infection condition [treatment]; which antibody was used for immunofluorescence [marker]; cell cycle stage [interphase (I), mitosis (M)]; whether cells within the group were considered to be infected (1, 4G2 positive) or not (0) [4G2+], note that only infected cells and mock treated cells were retained for further analysis; the number of cells in the group [n total]; the number of cells with more than 4 centriolar foci (POC5, CP110) or more than 2 centriolar foci (PolyE) [n overamplification]; the percentage of cells exhibiting an overamplification phenotype [% overamplification] within that group; the average angle of spindle positioning with respect to the fibronectin substratum [mean α]; the angle standard deviations [s.d. α]; which biological replicate this is

[replicate #]; the Figure in which the data is used [figure]. * Please note that in rows 72, 75, 78, 81, 84 and 87 cells of all stages of mitosis were scored for centriolar foci number, whereas only metaphase cells were scored for spindle positioning. This is indicated in the column [cell number], where the first number corresponds to all mitotic cells and the second to metaphase cells.

Supplemental Figure 1

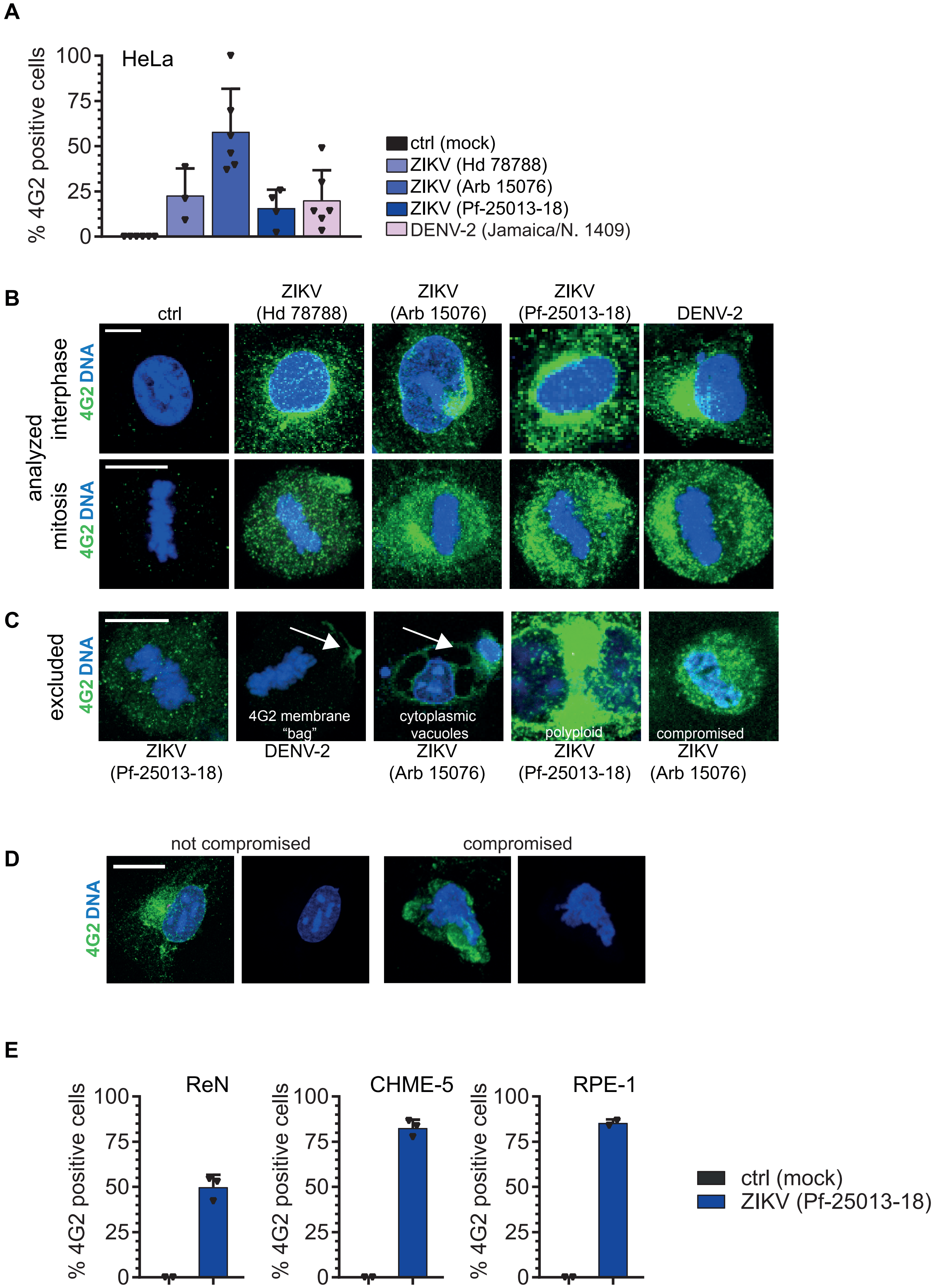

Supplemental Figure 2

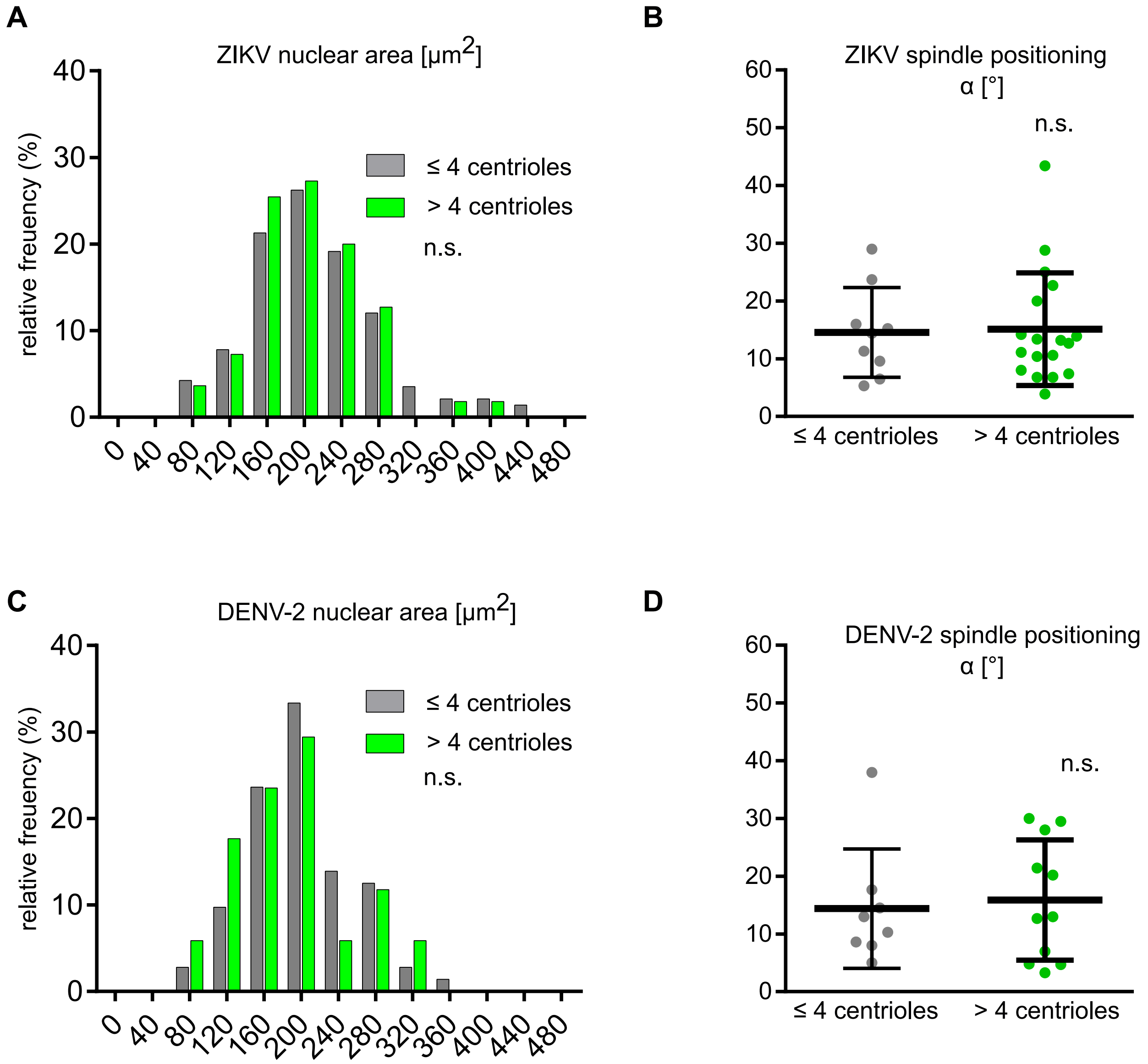

Table S1

cover-slip	cell	treatment	marker	inter-phase (I), mitosis (M)	4G2+	n total	n over-ampli-fication	% over-ampli-fication	mean α	s.d. α	repli-cate	figure
1	HeLa	mock	CP110	I	0	5	0	0			2	1D/ 4D
2	HeLa	mock	POC5	I	0	1	0	0			1	1D/ 4D
3	HeLa	mock	POC5	I	0	1	0	0			2	1D/ 4D
4	HeLa	mock	PolyE	I	0	30	4	13			1	1D/ 4D
5	HeLa	mock	PolyE	I	0	9	0	0			3	1D/ 4D
6	HeLa	mock	CP110	M	0	2	0	0	9.2	0.8	2	1G/ 3B/ 4D/ 4G
7	HeLa	mock	CP110	M	0	3	1	33	9.4	8.5	3	1G/ 3B/ 4D/ 4G
8	HeLa	mock	POC5	M	0	20	2	10	7.2	5.5	1	1G/ 3B/ 4D/ 4G
9	HeLa	mock	POC5	M	0	11	2	18	5.8	2.5	2	1G/ 3B/ 4D/ 4G
10	HeLa	mock	POC5	M	0	20	2	10	5.5	4.2	3	1G/ 3B/ 4D/ 4G
11	HeLa	mock	PolyE	M	0	20	0	0	5.2	3.7	1	1G/ 3B/ 4D/ 4G
12	HeLa	mock	PolyE	M	0	12	0	0	7.2	7.7	2	1G/ 3B/ 4D/ 4G
13	HeLa	mock	PolyE	M	0	20	1	5	5.9	4.5	3	1G/ 3B/ 4D/ 4G
14	HeLa	DENV-2	CP110	I	1	18	0	0			2	4D
15	HeLa	DENV-2	CP110	I	1	15	6	40			3	4D
16	HeLa	DENV-2	POC5	I	1	5	0	0			2	4D
17	HeLa	DENV-2	POC5	I	1	3	2	67			3	4D
18	HeLa	DENV-2	PolyE	I	1	27	10	37			1	4D
19	HeLa	DENV-2	CP110	M	0	7	1	14	8.9	3.8	2	
20	HeLa	DENV-2	CP110	M	0	1	0	0	7.6		3	
21	HeLa	DENV-2	CP110	M	1	1	0	0	12.4		2	4D/ 4G
22	HeLa	DENV-2	CP110	M	1	1	1	100	3.3		3	4D/ 4G
23	HeLa	DENV-2	POC5	M	0	13	6	46	9.7	6.3	1	
24	HeLa	DENV-2	POC5	M	0	18	3	17	9.6	5.4	2	
25	HeLa	DENV-2	POC5	M	0	11	3	27	8.3	4.7	3	
26	HeLa	DENV-2	POC5	M	1	3	2	67	8.5	4.0	1	4D/ 4G
27	HeLa	DENV-2	POC5	M	1	1	1	100	4.8		2	4D/ 4G
28	HeLa	DENV-2	POC5	M	1	1	0	0	14.5		3	4D/ 4G
29	HeLa	DENV-2	PolyE	M	0	18	10	56	7.6	4.6	1	
30	HeLa	DENV-2	PolyE	M	1	14	5	36	20.2	12.0	1	4D/ 4G
31	HeLa	DENV-2	PolyE	M	1	1	1	100	7.0		2	4D/ 4G
32	HeLa	ZIKV Hd 78788	CP110	I	1	21	5	24			1	1D
33	HeLa	ZIKV Hd 78788	CP110	I	1	12	3	25			2	1D
34	HeLa	ZIKV Hd 78788	POC5	I	1	3	1	33			2	1D
35	HeLa	ZIKV Hd 78788	PolyE	I	1	7	4	57			1	1D
36	HeLa	ZIKV Hd 78788	CP110	M	0	8	2	25	13.2	7.5	2	
37	HeLa	ZIKV Hd 78788	POC5	M	0	11	7	64	13.6	6.6	1	
38	HeLa	ZIKV Hd 78788	POC5	M	0	2	0	0	3.2	0.9	2	
39	HeLa	ZIKV Hd 78788	POC5	M	1	7	3	43	13.0	1.7	1	1G/ 3B
40	HeLa	ZIKV Hd 78788	PolyE	M	0	14	4	29	14.7	13.5	1	
41	HeLa	ZIKV Hd 78788	PolyE	M	0	4	1	25	12.8	8.9	2	
42	HeLa	ZIKV Hd 78788	PolyE	M	1	2	2	100	10.8	4.8	1	1G/ 3B
43	HeLa	ZIKV Hd 78788	CP110	M	1	10	4	40			2	1G/
44	HeLa	ZIKV Arb 15076	CP110	I	1	14	7	50			1	1D
45	HeLa	ZIKV Arb 15076	CP110	I	1	22	7	32			2	1D
46	HeLa	ZIKV Arb 15076	POC5	I	1	6	1	17			2	1D
47	HeLa	ZIKV Arb 15076	PolyE	I	1	8	1	13			1	1D
48	HeLa	ZIKV Arb 15076	PolyE	I	1	5	2	40			2	1D
49	HeLa	ZIKV Arb 15076	CP110	M	0	3	1	33	6.4	4.2	1	
50	HeLa	ZIKV Arb 15076	POC5	M	0	4	1	25	6.2	7.2	1	
51	HeLa	ZIKV Arb 15076	POC5	M	0	7	2	29	13.0	17.2	2	
52	HeLa	ZIKV Arb 15076	POC5	M	1	1	0	0	23.7		1	1G/ 3B
53	HeLa	ZIKV Arb 15076	POC5	M	1	8	4	50	9.7	5.6	2	1G/ 3B
54	HeLa	ZIKV Arb 15076	PolyE	M	0	4	0	0	13.7	7.0	1	
55	HeLa	ZIKV Arb 15076	PolyE	M	0	6	1	17	8.2	5.3	2	
56	HeLa	ZIKV Arb 15076	PolyE	M	1	3	2	67	27.4	22.7	1	1G/ 3B
57	HeLa	ZIKV Arb 15076	CP110	M	1	2	1	50				1G
58	HeLa	ZIKV PF-25013-18	CP110	I	1	9	2	22			2	1D
59	HeLa	ZIKV PF-25013-18	POC5	I	1	43	16	37			1	1D
60	HeLa	ZIKV PF-25013-18	POC5	I	1	8	3	38			2	1D
61	HeLa	ZIKV PF-25013-18	PolyE	I	1	9	4	44			1	1D
62	HeLa	ZIKV PF-25013-18	CP110	M	0	5	3	60	6.8	3.1	2	
63	HeLa	ZIKV PF-25013-18	POC5	M	0	20	11	55	12.4	6.9	1	
64	HeLa	ZIKV PF-25013-18	POC5	M	0	12	1	8	5.7	4.0	2	
65	HeLa	ZIKV PF-25013-18	POC5	M	1	2	1	50	32.9	5.7	1	1G/ 3B
66	HeLa	ZIKV PF-25013-18	POC5	M	1	3	2	67	15.4	12.7	2	1G/ 3B

67	HeLa	ZIKV PF-25013-18	PolyE	M	0	15	0	0	7.9	4.6	1	
68	HeLa	ZIKV PF-25013-18	PolyE	M	0	7	0	0	7.8	5.6	2	
69	HeLa	ZIKV PF-25013-18	PolyE	M	1	4	2	50	17.7	6.5	1	1G/ 3B
70	HeLa	ZIKV PF-25013-18	PolyE	M	1	2	0	0	14.8	0.6	2	1G/ 3B
71	RPE-1	mock	CP110	M	0	53	2	4			1	2B
72	RPE-1	mock	CP110	M	0	*39/3	1	3	5.3	1.1	1	2B/ 3D
73	RPE-1	mock	POC5	M	0	43	1	2			1	2B
74	RPE-1	ZIKV PF-25013-18	CP110	M	1	19	2	11			1	2B
75	RPE-1	ZIKV PF-25013-18	CP110	M	1	*36/9	5	22	12.7	7.1	1	2B/ 3D
76	RPE-1	ZIKV PF-25013-18	POC5	M	1	18	3	17			1	2B
77	CHME-5	mock	CP110	M	0	47	2	4			1	2D
78	CHME-5	mock	CP110	M	0	*40/29	1	3	6.1	3.9	1	2D/ 3F
79	CHME-5	mock	POC5	M	0	48	4	9			1	2D
80	CHME-5	ZIKV PF-25013-18	CP110	M	1	57	14	25			1	2D
81	CHME-5	ZIKV PF-25013-18	CP110	M	1	*41/27	9	22	15.4	11.5	1	2D/ 3F
82	CHME-5	ZIKV PF-25013-18	POC5	M	1	54	7	13			1	2D
83	ReN	mock	CP110	M	0	50	0	0			1	2F
84	ReN	mock	P110/ Poly	M	0	*19/13	0	0	9.9	5.7	1	2F / 3H
85	ReN	mock	POC5	M	0	25	0	0			1	2F
86	ReN	ZIKV PF-25013-18	CP110	M	1	23	0	0			1	2F
87	ReN	ZIKV PF-25013-18	P110/ Poly	M	1	*18/10	1	8	12.5	8.8	1	2F / 3H
88	ReN	ZIKV PF-25013-18	POC5	M	1	12	2	17			1	2F
